# A multinomial quadrivariate D-vine copula mixed model for meta-analysis of diagnostic studies in the presence of non-evaluable subjects

**DOI:** 10.1177/0962280220913898

**Published:** 2020-04-23

**Authors:** Aristidis K Nikoloulopoulos

**Affiliations:** School of Computing Sciences, University of East Anglia, Norwich, UK

**Keywords:** Diagnostic tests, multivariate meta-analysis, sensitivity, specificity, summary receiver operating characteristic curves

## Abstract

Diagnostic test accuracy studies observe the result of a gold standard procedure that defines the presence or absence of a disease and the result of a diagnostic test. They typically report the number of true positives, false positives, true negatives and false negatives. However, diagnostic test outcomes can also be either non-evaluable positives or non-evaluable negatives. We propose a novel model for the meta-analysis of diagnostic studies in the presence of non-evaluable outcomes, which assumes independent multinomial distributions for the true and non-evaluable positives, and, the true and non-evaluable negatives, conditional on the latent sensitivity, specificity, probability of non-evaluable positives and probability of non-evaluable negatives in each study. For the random effects distribution of the latent proportions, we employ a drawable vine copula that can successively model the dependence in the joint tails. Our methodology is demonstrated with an extensive simulation study and applied to data from diagnostic accuracy studies of coronary computed tomography angiography for the detection of coronary artery disease. The comparison of our method with the existing approaches yields findings in the real data application that change the current conclusions.

## 1 Introduction

Diagnostic test accuracy studies observe the result of a gold standard procedure that defines the presence or absence of a disease and the result of a diagnostic test. They typically report the number of true positives (diseased subjects correctly diagnosed), false positives (non-diseased subjects incorrectly diagnosed as diseased), true negatives (non-diseased subjects correctly diagnosed as non-diseased) and false negatives (diseased subjects incorrectly diagnosed as non-diseased). However, diagnostic test outcomes can be non-evaluable.^1^ This is the case for coronary computed tomography (CT) angiography studies which have non-evaluable results of index text in various ways such as when transferring a segment/vessel to a patient based evaluation.^2^

Synthesis of diagnostic test accuracy studies is the most common medical application of multivariate meta-analysis.^3,4^ The purpose of a meta-analysis of diagnostic test accuracy studies is to combine information over different studies and provide an integrated analysis that will have more statistical power to detect an accurate diagnostic test than an analysis based on a single study. Nevertheless, the existence of non-evaluable subjects is an important issue that could lead to biased meta-analytic estimates of index test accuracy.^2,5,6^ Schuetz et al.^2^ studied different ad-hoc approaches dealing with diagnostic test non-evaluable subjects, such as non-evaluable subjects are excluded from the study, non-evaluable positives (non-evaluable diseased subjects) are taken as true positives and non-evaluable negatives (non-evaluable non-diseased subjects) are taken as false positives, non-evaluable positives are taken as false negatives and non-evaluable negatives are taken as true negatives, and non-evaluable positives as false negatives and non-evaluable negatives as false positives. In all of these approaches, Schuetz et al.^2^ used the bivariate generalized linear mixed model (BGLMM)^7^ and concluded that excluding the index test non-evaluable subjects leads to overestimation of the meta-analytic estimates of sensitivity and specificity and recommended the intent-to-diagnose approach by treating non-evaluable positives as false negatives and non-evaluable negatives as false positives.

Ma et al.^5^ used a trivariate generalized mixed model (TGLMM) approach by treating the non-evaluable subjects as missing data under a missing at random assumption (MAR). Ma et al.,^5^ with extensive simulation studies, showed that the intent-to-diagnose approach^2^ under-estimates both meta-analytic estimates of sensitivity and specificity, while the TGLMM approach under the MAR assumption gives nearly unbiased estimates of sensitivity, specificity and prevalence.

Nikoloulopoulos,^6^ similar to Ma et al.,^5^ extended the vine copula mixed model for trivariate meta-analysis of diagnostic test accuracy studies accounting for disease prevalence^8^ to additionally account for non-evaluable subjects. The extended trivariate vine copula mixed model includes the extended TGLMM as a special case and can also model sensitivity, specificity and prevalence on the original scale. Nikoloulopoulos^6^ demonstrated that the extended TGLMM leads to biased meta-analytic estimates of sensitivity, specificity and prevalence when the univariate random effects are misspecified and that the extended vine copula mixed model gives nearly unbiased estimates of test accuracy indices and disease prevalence.

A recurrent theme underlying the methodologies of Ma et al.^5^ and Nikoloulopoulos^6^ is the need to make the MAR assumption that cannot be verified based on the observed data. Hence, it is natural to be concerned about robustness or sensitivity of inferences to departures from the MAR assumption. The within-study model assumes that the number of true negatives, false negatives, false positives, true positives, non-evaluable negatives and non-evaluable positives are multinomially distributed, given the latent (random) vector of sensitivity, specificity, disease prevalence, probability of non-evaluable positives and probability of non-evaluable negatives. Under the MAR assumption, the multinomial probability mass function (pmf) decomposes into a product of independent binomial pmfs given the random effects. Hence, the within-study model actually assumes that the number of true negatives, number of true positives, number of diseased subjects, number of non-evaluable negatives and number of non-evaluable positives are conditionally independent and binomially distributed given the random effects. The triplet of latent sensitivity, specificity and prevalence are independent of the missing probabilities, hence the joint likelihood factors into two components, one involving only the sensitivity, specificity and disease prevalence, and the other involving only the probabilities of non-evaluable positives and non-evaluable negatives. Therefore, the methodology of Chu et al.^9^ or Nikoloulopoulos^8^ is applied to the first likelihood component to infer about the sensitivity, specificity and disease prevalence. Hence, the models in Ma et al.^5^ and Nikoloulopoulos^6^ extend the BGLMM^7^ and the bivariate vine copula mixed model,^10^ respectively, to the trivariate case by adding the disease prevalence as a third outcome to indirectly account for the non-evaluable results. On the one hand, the number of diseased subjects are binomially distributed with probability of success the latent prevalence and a support that includes the number of non-evaluable positives and the number of non-evaluable negatives, but the true positives and true negatives are binomially distributed with probability of success the latent sensitivity and specificity, respectively, and a support that does not include either the number of non-evaluable positives or the number of non-evaluable negatives on the other, just like in the BGLMM^7^ and the bivariate vine copula mixed model.^10^ Note in passing that a special case of the bivariate copula mixed model is the BGLMM, that is, a copula mixed model composed of a bivariate normal (BVN) copula with normal margins.

In this paper, in order to remedy this situation of ignoring the non-evaluable subjects in the derivation of the meta-analytic estimates of sensitivity and specificity, we include the number of non-evaluable positives and the number of non-evaluable negatives as separate non-missing response categories. Interestingly, the proposed model extends the bivariate copula mixed model^10^ to the quadrivariate case by directly adding the number of non-evaluable positives and number of non-evaluable negatives as a third and fourth outcome, respectively. Hence, it directly utilizes all the available data. The bivariate copula mixed model^10^ assumes independent binomial distributions for the true positives and true negatives, conditional on the latent pair of sensitivity and specificity in each study. In the proposed methodology for the meta-analysis of diagnostic tests where we additionally account for non-evaluable outcomes of the diagnostic test, we will assume independent multinomial distributions for the true and non-evaluable positives, and, the true and non-evaluable negatives, conditional on the latent sensitivity, specificity, probability of non-evaluable positives and probability of non-evaluable negatives in each study.

For the random effects distribution, we employ a regular vine copula.^11^ Regular vine copulas are suitable for high-dimensional data, hence given the low dimension *d *=* *4, where *d* is the dimension, we use their boundary case namely a drawable vine (D-vine) copula. D-vine copulas have become important in many applications areas such as finance^12,13^ and biological sciences,^14,15^ to just name a few, in order to deal with dependence in the joint tails. Another boundary case of regular vine copulas is the canonical vine copula, but this parametric family of copulas is only suitable if there exists a (pilot) variable that drives the dependence among the variables,^16,17^ which apparently is not the case in this application area.

The remainder of the paper proceeds as follows. Section 2 introduces the multinomial quadrivariate D-vine copula mixed model for meta-analysis of diagnostic studies accounting for non-evaluable results and provides computational details for maximum likelihood (ML) estimation. Section 3 studies the small-sample efficiency and robustness of the ML estimation of the multinomial quadrivariate D-vine copula mixed model. Section 4 applies our methodology to data from a published meta-analysis for diagnostic accuracy studies of coronary computed tomography angiography for the detection of coronary artery disease. We conclude with some discussion in Section 5, followed by a brief section with software details.

## 2 The multinomial quadrivariate D-vine copula mixed model

In this section, we introduce the multinomial quadrivariate D-vine copula mixed model. In Subsections 2.1 and 2.2, a D-vine copula representation of the random effects distribution with normal and beta margins, respectively, is presented. We complete this section with details on maximum likelihood estimation.

### 2.1 The multinomial quadrivariate D-vine copula mixed model with normal margins

We first introduce the notation used in this paper. The data are $y_{\mathrm{ijk}},\;i=1,\ldots ,N,\;j=0,\;1,\;2,\;k=0,1$, where *i* is an index for the individual studies, *j* is an index for the test outcome (0: negative; 1: positive; 2: non-evaluable) and *k* is an index for the disease outcome (0: non-diseased; 1: diseased). The “classic” 2 × 2 table is extended to a 3 × 2 table (Table 1). Each cell in Table 1 provides the cell frequency corresponding to a combination of index test and disease outcomes in study *i*.

**Table 1.** Data from an individual study in a 3 × 2 table.

	Disease (by gold standard)		
Test	–	+	Total
–	$y_{i00}$	$y_{i01}$	$y_{i0+}$
+	$y_{i10}$	$y_{i11}$	$y_{i1+}$
Non-evaluable	$y_{i20}$	$y_{i21}$	$y_{i2+}$
Total	$y_{i+0}$	$y_{i+1}$	$y_{i++}$

The diseased subjects have three possible states: false negative, true positive, and non-evaluable positive. The multinomial observation is therefore the number of diseased subjects where the diagnostic test is in each of its states. Hence, we assume that the false negatives $Y_{i01}$, the true positives $Y_{i11}$, and the non-evaluable positives $Y_{i21}$ are multinomially distributed given $\left(X_{1}=x_{1},X_{3}=x_{3}\right)$, viz.

(1) (Yi01, Yi11, Yi21)|(X1=x1, X3=x3) ∼M3(yi+1, 1−l−1(x1, x3)−l−1(x3, x1), l−1(x1, x3), l−1(x3, x1))

where (*X*_1_, *X*_3_) is the bivariate latent pair of transformed sensitivity and probability of non-evaluable positives and $l^{-1}\left(x_{j},\;x_{k}\right)=\frac{e^{x_{j}}}{1+e^{x_{j}}+e^{x_{k}}}$ is the inverse multinomial logit link. Note that $M_{T}(n,\;p_{1},\ldots ,p_{T})$ is shorthand notation for the multinomial distribution, where *T* is the number of cells, *n* is the number of observations, and $\left(p_{1},\ldots ,p_{T}\right)$ with $p_{1}+\cdots +p_{T}=1$ is the *T*-dimensional vector of success probabilities.

In a similar manner, the non-diseased subjects have also three possible states: true negative, false positive, and non-evaluable negative. Hence, we assume that the true negatives $Y_{i00}$, the false positives $Y_{i10}$, and the non-evaluable negatives $Y_{i20}$ are multinomially distributed given $\left(X_{2}=x_{2},X_{4}=x_{4}\right)$, viz.

(2) (Yi00, Yi10, Yi20)|(X2=x2, X4=x4) ∼M3(yi+0, l−1(x2, x4), 1−l−1(x2, x4)−l−1(x4, x2), l−1(x4, x2))

where (*X*_2_, *X*_4_) is the bivariate latent pair of transformed specificity and probability of non-evaluable negatives.

After defining the within-studies model in equations (1) and (2), we next define the between-studies model. The stochastic representation of the between studies model takes the form

(3) (Φ(X1; l(π1, π3), σ12), Φ(X2; l(π2, π4), σ22), Φ(X3; l(π3, π1), σ32), Φ(X4; l(π4, π2), σ42))∼C(·;θ)

where C(·;θ) is a quadrivariate D-vine copula with dependence parameter vector $\theta =\left(\theta_{12},\;\theta_{23},\;\theta_{34},\;\theta_{13|2},\;\theta_{24|3},\;\theta_{14|23}\right)$ and $\Phi \left(\cdot ;\mu ,\sigma^{2}\right)$ is the cumulative distribution function (cdf) of the N($\mu ,\sigma^{2}$) distribution, and $l\left(\pi_{j},\;\pi_{k}\right)=\log\left(\frac{\pi_{j}}{1-\pi_{j}-\pi_{k}}\right)$ is the multinomial logit link. The copula parameter vector θ has parameters of the random effects model and they are separated from the univariate parameters $(\pi_{j},\sigma_{j}),\;j=1,\ldots ,4$. The parameters *π*_1_ and *π*_2_ are those of actual interest denoting the meta-analytic parameters for the sensitivity and specificity, while the parameters *π*_3_ and *π*_4_ denote the probabilities of non-evaluable positives and negatives, respectively. The univariate parameters $\sigma_{1}^{2},\sigma_{2}^{2},\sigma_{3}^{2},\sigma_{4}^{2}$ denote the variabilities of the random effects.

The quadrivariate D-vine copula is built via successive mixing from bivariate pair-copulas on different levels. The pairs at level 1 are j,j+1, for *j *=* *1, 2, 3, and for level ℓ (2≤ℓ<4), the (conditional) pairs are j,j+ℓ|j+1,…,j+ℓ−1 for j=1,…,4−ℓ. That is, for the four-dimensional D-vine, the copulas for variables *j* and j+ℓ given the variables indexed in between capture the conditional dependence.^13^ When all the bivariate pair-copulas are BVN copulas with correlation (copula) parameters $\rho_{12},\rho_{23},\rho_{34}$ (1st level) and partial correlation parameters $\rho_{13|2},\rho_{24|3},\rho_{14|23}$ (2nd and 3rd level), the resulting distribution is the quadrivariate normal with mean vector $\mu ={\left(l\left(\pi_{1},\;\pi_{3}\right),\;l\left(\pi_{2},\;\pi_{3}\right),\;l\left(\pi_{3},\;\pi_{1}\right),\;l\left(\pi_{4},\;\pi_{2}\right)\right)}^{⊤}$ and variance covariance matrix

$$\sum =\left(\begin{array}{cccc} \sigma_{1}^{2} & \rho_{12}\sigma_{1}\sigma_{2} & \rho_{13}\sigma_{1}\sigma_{3} & \rho_{14}\sigma_{1}\sigma_{4}\\ \rho_{12}\sigma_{1}\sigma_{2} & \sigma_{2}^{2} & \rho_{23}\sigma_{2}\sigma_{3} & \rho_{24}\sigma_{2}\sigma_{4}\\ \rho_{13}\sigma_{1}\sigma_{3} & \rho_{23}\sigma_{2}\sigma_{3} & \sigma_{3}^{2} & \rho_{34}\sigma_{3}\sigma_{4}\\ \rho_{14}\sigma_{1}\sigma_{4} & \rho_{24}\sigma_{2}\sigma_{4} & \rho_{34}\sigma_{3}\sigma_{4} & \sigma_{4}^{2} \end{array}\right)$$

where $\rho_{13}=\rho_{13|2}\sqrt{1-\rho_{12}^{2}}\sqrt{1-\rho_{23}^{2}}+\rho_{12}\rho_{23}$, $\rho_{24}=\rho_{24|3}\sqrt{1-\rho_{23}^{2}}\sqrt{1-\rho_{34}^{2}}+\rho_{23}\rho_{34}$, $\rho_{14}=\rho_{14|2}\sqrt{1-\rho_{12}^{2}}\sqrt{1-\rho_{24}^{2}}+\rho_{12}\rho_{24}$, $\rho_{14|2}=\rho_{14|23}\sqrt{1-\rho_{13|2}^{2}}\sqrt{1-\rho_{34|2}^{2}}+\rho_{13|2}\rho_{34|2}$, $\rho_{13|2}=(\rho_{13}-\rho_{12}\rho_{23})/\sqrt{1-\rho_{12}^{2}}/\sqrt{1-\rho_{23}^{2}}$ and $\rho_{34|2}=(\rho_{34}-\rho_{23}\rho_{24})/\sqrt{1-\rho_{23}^{2}}/\sqrt{1-\rho_{24}^{2}}$.^15^ Other choices of copulas are better if there is more dependence in joint upper or lower tail.

The models in equations (1)–(3) together specify a multinomial quadrivariate D-vine copula mixed model with joint likelihood

L(π1, π2, π3, π4, σ1, σ2, σ3, σ4, θ)=∏i=1N∫−∞∞∫−∞∞∫−∞∞∫−∞∞g(yi11, yi21; yi+1, l−1(x1, x3), l−1(x3, x1)) ×g(yi00, yi20; yi+0, l−1(x2, x4), l−1(x4, x2))f1234(x1, x2, x3, x4; θ) dx1 dx2 dx3 dx4

where $g(;n,p_{1},\ldots ,p_{T-1})$ is the $M_{T}\left(n,\;p_{1},\ldots ,p_{T}\right)$ pmf and $f_{1234}(\cdot ;\theta )$ is the quadrivariate D-vine density, viz.

(4) $$f_{1234}\left(x_{1},x_{2},x_{3},x_{4};\theta \right)=\phi (x_{1})\phi (x_{2})\phi (x_{3})\phi (x_{4})c_{1234}\left(\Phi (x_{1}),\Phi (x_{2}),\Phi (x_{3}),\Phi (x_{4});\theta \right)$$

with

c1234(Φ(x1),Φ(x2),Φ(x3),Φ(x4);θ)=c12(Φ(x1),Φ(x2);θ12)c23(Φ(x2),Φ(x3);θ23)c34(Φ(x3),Φ(x4);θ34) ×c13|2(F1|2(x1|x2),F3|2(x3|x2);θ13|2)c24|3(F2|3(x2|x3),F4|3(x4|x3);θ24|3) ×c14|23(F1|23(x1|x2,x3),F4|23(x4|x2,x3);θ14|23)

where ϕ(x) and Φ(x) is shorthand notation for the density $\phi (x;\mu ,\sigma^{2})$ and cdf $\Phi (x;\mu ,\sigma^{2})$ of the $N(\mu ,\sigma^{2})$ distribution, $c_{jk},c_{jk|\ell },c_{14|23}$ are bivariate copula densities, $F_{j|k}\left(x_{j}|x_{k}\right)=\frac{\partial C_{jk}\left(\Phi_{j}(x_{j}),\;\Phi_{k}(x_{k})\right)}{\partial \Phi_{k}(x_{k})}$, $F_{1|23}\left(x_{1}|x_{2},\;x_{3}\right)=\frac{\partial C_{13|2}\left(F_{1|2}(x_{1}|x_{2}),\;F_{3|2}(x_{3}|x_{2})\right)}{\partial \Phi (x_{2})}$ and $F_{4|23}\left(x_{4}|x_{2},x_{3}\right)=\frac{\partial C_{24|3}\left(F_{2|3}(x_{2}|x_{3}),\;F_{4|3}(x_{4}|x_{3})\right)}{\partial \Phi (x_{3})}$; $C_{jk},C_{jk|\ell }$ are bivariate copula cdfs. Note that a for a four-dimensional D-vine copula density there are 12 different decompositions.^12^ To be concrete in the exposition of the theory, we use the decomposition in equation (4); the theory though also applies to the other 11 decompositions.

Below we transform the original integral into an integral over a unit hypercube using the inversion method. Hence, the joint likelihood becomes

∏i=1N∫01∫01∫01∫01g(yi11,yi21; yi+1, l−1(Φ−1(u1, l(π1, π3), σ12), Φ−1(u3, l(π3,π1), σ32)), l−1(Φ−1(u3, l(π3, π1), σ32), Φ−1(u1, l(π1, π3), σ12))) ×g(yi00,yi20;yi+0,l−1(Φ−1(u2, l(π2, π4), σ22), Φ−1(u4, l(π4, π2), σ42)), l−1(Φ−1(u4, l(π4, π2), σ42), Φ−1(u2, l(π2, π4), σ22)))c1234(u1, u2, u3, u4; θ) du

### 2.2 The multinomial D-vine copula mixed model with beta margins

In this section, we use the parametrization proposed by Wilson^18^ in order the latent sensitivity and specificity to remain on the original scale. The within-study model takes the form

(5) (Yi01, Yi11, Yi21|(X1=x1,X3=x3)∼M3(yi+1, 1−x1−x3(1−x1), x1, x3(1−x1));(Yi00, Yi10, Yi20|(X2=x2,X4=x4)∼M3(yi+0,x2, 1−x2−x4(1−x2), x4(1−x2))

The stochastic representation of the between studies model is

(6) $$\left(F\left(X_{1};\;\pi_{1},\;\gamma_{1}\right),\;F\left(X_{2};\;\pi_{2},\;\gamma_{2}\right),\;F\left(X_{3};\;\frac{\pi_{3}}{1-\pi_{1}},\;\gamma_{3}\right),\;F\left(X_{4};\;\frac{\pi_{4}}{1-\pi_{2}},\;\gamma_{4}\right)\right)∼C\left(\cdot ;\theta \right)$$

where C(·;θ) is a D-vine copula with dependence parameter vector θ and F(·;π,γ) is the cdf of the Beta(π,γ) distribution with *π* the mean and *γ* the dispersion parameter. The copula parameter vector θ has the dependence parameters of the random effects model and they are separated from the univariate parameters $(\pi_{j},\gamma_{j}),\;j=1,\ldots ,4$. The parameters *π*_1_ and *π*_2_ are those of actual interest denoting the meta-analytic parameters for the sensitivity and specificity, while the parameters *π*_3_ and *π*_4_ denote the probabilities of non-evaluable positives and negatives, respectively. The univariate parameters $\gamma_{1},\gamma_{2},\gamma_{3},\gamma_{4}$ denote the variabilities of the random effects. In contrast with the model in the preceding subsection, the random effects of sensitivity and specificity are on the original scale.

The models in equations (5) and (6) together specify a vine copula mixed model with joint likelihood

L(π1, π2, π3, π4, γ1, γ2, γ3, γ4, θ)=∏i=1N∫01∫01∫01∫01g(yi11, yi21; yi+1, x1, x3(1−x1))g(yi00, yi02; yi+0, x2, x4(1−x2)) ×f1234(x1, x2, x3, x4; θ) dx1 dx2 dx3 dx4

where $f_{1234}(\cdot ;\theta )$ is as in equation (4) where we use beta instead of normal marginal distributions. Below we transform the integral into an integral over a unit hypercube using the inversion method. Hence, the joint likelihood becomes

∏i=1N∫01∫01∫01∫01g(yi11, yi21; yi+1, F−1(u1;π1, γ1), F−1(u3;π31−π1,γ3)(1−F−1(u1; π1, γ1))) ×g(yi00,yi20;yi+0,F−1(u2; π2, γ2), F−1(u4; π41−π2, γ4)(1−F−1(u2;π2,γ2))) ×c1234(u1, u2, u3, u4; θ) du1 du2 du3 du4

### 2.3 Maximum likelihood estimation and computational details

Estimation of the model parameters can be approached by the standard maximum likelihood (ML) method, by maximizing the logarithm of the joint likelihood. The estimated parameters can be obtained by using a quasi-Newton^19^ method applied to the logarithm of the joint likelihood. This numerical method requires only the objective function, i.e. the logarithm of the joint likelihood, while the gradients are computed numerically and the Hessian matrix of the second-order derivatives is updated in each iteration. The standard errors (SEs) of the ML estimates can be also obtained via the gradients and the Hessian computed numerically during the maximization process.

For the multinomial quadrivariate D-vine copula mixed model, numerical evaluation of the joint pmf can be achieved with the following steps:

1. Calculate Gauss-Legendre^20^ quadrature points $\left\{u_{q}:q=1,\ldots ,n_{q}\right\}$ and weights $\left\{w_{q}:q=1,\ldots ,n_{q}\right\}$ in terms of standard uniform.
2. Convert from independent uniform random variables $\left\{u_{q_{1}}:q_{1}=1,\ldots ,n_{q}\right\},\;\left\{u_{q_{2}}:q_{2}=1,\ldots ,n_{q}\right\},\left\{u_{q_{3}}:q_{3}=1,\ldots ,n_{q}\right\}$, and $\left\{u_{q_{4}}:q_{4}=1,\ldots ,n_{q}\right\}$ to dependent uniform random variables $v_{q_{1}},v_{q_{2}|q_{1}},v_{q_{3}|q_{1};q_{2}}$, and $v_{q_{4}|q_{1};q_{2},q_{3}}$ that have a D-vine distribution C(·;θ) using the algorithm in Nikoloulopoulos^15^:
  1. Set $v_{q_{1}}=u_{q_{1}}$
  2. $v_{q_{2}|q_{1}}=C_{2|1}^{-1}(u_{q_{2}}|u_{q_{1}};\theta_{12})$
  3. $t_{1}=C_{1|2}(v_{q_{1}}|v_{q_{2}|q_{1}};\theta_{12})$
  4. $t_{2}=C_{3|1;2}^{-1}\left(u_{q_{3}}|t_{1};\theta_{12}),\theta_{13|2}\right)$
  5. $v_{q_{3}|q_{1};q_{2}}=C_{3|2}^{-1}(t_{2}|v_{q_{2}|q_{1}};\theta_{23})$
  6. $t_{3}=C_{2|3}(v_{q_{2}|q_{1}}|v_{q_{3}|q_{1};q_{2}};\theta_{23})$
  7. $t_{4}=C_{1|3;2}(t_{1}|t_{2};\theta_{13|2})$
  8. $t_{5}=C_{4|1;2,3}(u_{q_{4}}|t_{4};\theta_{14|23})$
  9. $t_{6}=C_{4|2;3}^{-1}(t_{5}|t_{3};\theta_{24|3})$
  10. $v_{q_{4}|q_{1};q_{2},q_{3}}=C_{4|3}^{-1}(t_{6}|v_{q_{3}|q_{1};q_{2}};\theta_{34})$

where C(v|u;θ) and $C^{-1}(v|u;\theta )$ are conditional copula cdfs and their inverses.

3. Numerically evaluate the joint pmf, e.g.
  ∏i=1N∫01∫01∫01∫01g(yi11,yi21;yi+1,F−1(u1;π1,γ1), F−1(u3; π31−π1, γ3)(1−F−1(u1; π1, γ1))) ×g(yi00, yi20; yi+0, F−1(u2; π2, γ2), F−1(u4; π41−π2, γ4)(1−F−1(u2;π2,γ2))) ×c1234(u1,u2,u3,u4;θ) du1 du2 du3 du4

in a quadruple sum

∑q1=1nq∑q2=1nq∑q3=1nq∑q4=1nqwq1wq2wq3wq4g(yi11,yi21; yi+1, F−1(vq1; π1,γ1), F−1(vq3|q1;q2; π31−π1, γ3)(1−F−1(vq1;π1,γ1)))g(yi00,yi20;yi+0, F−1(vq2|q1; π2, γ2), F−1(vq4|q1;q2,q3; π41−π2, γ4)(1−F−1(vq2|q1;π2, γ2)))

With Gauss-Legendre quadrature, the same nodes and weights are used for different functions; this helps in yielding smooth numerical derivatives for numerical optimization via quasi-Newton.

## 3 Simulations

In this section, we study the small-sample efficiency and robustness of the ML estimation of the multinomial quadrivariate D-vine copula mixed model. In Section 3.1, we gauge the small-sample efficiency of the ML method and investigate the misspecification of the parametric margin or bivariate pair-copulas of the random effects distribution. In Section 3.2, we investigate the mixed model misspecification by using both the proposed model and the extended trivariate vine copula mixed model^6^ as true models.

We set the sample size and the true univariate and dependence parameters to mimic the data analyzed in Section 4. In each model, we use six different linking copula families: normal, Frank, and Clayton copula along with its rotated versions (see our previous papers on copula mixed models^8,10,21,22^ for definitions) to cover different types of dependence structure. To make it easier to compare strengths of dependence, we convert the BVN, Frank, and rotated Clayton estimated parameters to Kendall’s *τ*’s in (−1,1) via the following relations^23,24^

τ=2πarcsin(θ)τ={1−4θ−1−4θ−2∫θ0tet−1 dt,θ<01−4θ−1+4θ−2∫0θtet−1 dt,θ>0

and^25^

$$\tau =\{\begin{array}{cc} \theta /\left(\theta +2\right), & \text{by}\;0{}^{\circ}\;\text{or}\;\;180{}^{\circ}\\ -\theta /\left(\theta +2\right), & \text{by}\;90{}^{\circ}\;\text{or}\;\;270{}^{\circ} \end{array}$$

### 3.1 Small-sample efficiency–misspecification of the random effects distribution

We randomly generate samples of size *N *=* *30 from the multinomial quadrivariate D-vine copula mixed model with both normal and beta margins. The simulation process is as below:

1. Simulate $\left(u_{1},u_{2},u_{3},u_{4}\right)$ from a D-vine distribution $C(\cdot ;\tau_{12},\tau_{23},\tau_{34},\tau_{13|2}=0,\tau_{24|3}=0,\tau_{14|23}=0)$.
2. • Convert to normal realizations via
  $$\begin{array}{ll} x_{1}=\Phi^{-1}\left(u_{1};\log\frac{\pi_{1}}{1-\pi_{1}-\pi_{3}},\;\sigma_{1}\right) & x_{2}=\Phi^{-1}\left(u_{2};\log\frac{\pi_{2}}{1-\pi_{2}-\pi_{4}},\;\sigma_{2}\right)\\ x_{3}=\Phi^{-1}\left(u_{3};\log\frac{\pi_{3}}{1-\pi_{1}-\pi_{3}},\;\sigma_{3}\right) & x_{4}=\Phi^{-1}\left(u_{4};\log\frac{\pi_{4}}{1-\pi_{2}-\pi_{4}},\;\sigma_{4}\right) \end{array}$$
 • Convert to beta realizations via
  $$\begin{array}{ll} x_{1}=F^{-1}\left(u_{1};\;\pi_{1},\;\gamma_{1}\right) & x_{2}=F^{-1}\left(u_{2};\;\pi_{2},\;\gamma_{2}\right)\\ x_{3}=F^{-1}\left(u_{3};\;\frac{\pi_{3}}{1-\pi_{1}},\;\sigma_{1}\right) & x_{4}=F^{-1}\left(u_{4};\;\log\frac{\pi_{4}}{1-\pi_{2}},\;\gamma_{4}\right) \end{array}$$
3. Simulate the size of diseased and non-diseased subjects *n*_1_ and *n*_2_, respectively, from a shifted gamma distribution to obtain heterogeneous study sizes,^26^ i.e.
  n1∼sGamma(α=1.2,β=0.01,lag=30)n2∼sGamma(α=1.2,β=0.01,lag=30)

and round off *n*_1_ and *n*_2_ to the nearest integers.

4. • For normal margins, draw $\left(y_{01},y_{11},y_{21}\right)$ from
  $$M_{3}\left(n_{1},\;\frac{1}{1+e^{x_{1}}+e^{x_{3}}},\;\frac{e^{x_{1}}}{1+e^{x_{1}}+e^{x_{3}}},\;\frac{e^{x_{3}}}{1+e^{x_{1}}+e^{x_{3}}}\right)$$
  and $\left(y_{00},y_{10},y_{20}\right)$ from
  $$M_{3}\left(n_{2},\;\frac{e^{x_{2}}}{1+e^{x_{2}}+e^{x_{4}}},\;\frac{1}{1+e^{x_{2}}+e^{x_{4}}},\;\frac{e^{x_{4}}}{1+e^{x_{2}}+e^{x_{4}}}\right)$$
 • For beta margins, draw $\left(y_{01},y_{11},y_{21}\right)$ from
  $$M_{3}\left(n_{1},\;1-x_{1}-x_{3}\left(1-x_{1}\right),\;x_{1},\;x_{3}\left(1-x_{1}\right)\right)$$

and $\left(y_{00},y_{10},y_{20}\right)$ from

$$M_{3}\left(n_{2},\;x_{2},\;1-x_{2}-x_{4}\left(1-x_{2}\right),\;x_{4}\left(1-x_{2}\right)\right)$$

Tables 2 and 3 contain the resultant biases, root mean square errors (RMSE), and standard deviations (SD), along with the square root of the average theoretical variances ($\sqrt{\bar{V}}$), scaled by 100, for the ML estimates under different pair-copulas and marginal choices from the multinomial D-vine copula mixed model with beta and normal margins, respectively. The true (simulated) pair-copula distributions are the Clayton copulas rotated by 180° for both the $C_{12}(;\tau_{12})$ and $C_{34}(;\tau_{34})$ pair-copulas and the Clayton copula rotated by 90° for the $C_{23}(;\tau_{23})$ pair-copula.

**Table 2.** Small sample of sizes *N *=* *30 simulations (10^3^ replications; *n_q_* = 15) from the multinomial quadrivariate D-vine copula mixed model with beta margins and resultant biases, root mean square errors (RMSE) and standard deviations (SD), along with the square root of the average theoretical variances ($\sqrt{\bar{V}}$), scaled by 100, for the ML estimates under different pair-copula choices and margins.

	Margin	Copula	$\pi_{1}$ = 0.90	$\pi_{2}=$0.77	$\pi_{3}$ = 0.06	$\pi_{4}$ = 0.11	$\gamma_{1}$ = 0.09	$\gamma_{2}$ = 0.08	$\gamma_{3}$ = 0.37	$\gamma_{4}$ = 0.15	$\tau_{12}$ = 0.82	$\tau_{23}$ = –0.52	$\tau_{34}$ = 0.26
Bias	Normal	BVN	4.20	3.49	–1.97	–1.91	–	–	–	–	–22.37	36.27	16.07
	Beta		–0.08	–0.03	0.38	0.03	–0.10	–0.21	–4.81	–0.12	–5.01	6.21	1.97
	Normal	Frank	4.24	3.68	–1.96	–1.86	–	–	–	–	–21.28	34.84	15.74
	Beta		0.21	0.43	0.11	–0.18	–0.01	–0.17	–4.25	–0.09	–2.58	5.14	2.00
	Normal	Cln{180°, 90°}	4.20	3.37	–2.00	–1.84	–	–	–	–	–15.90	30.22	15.57
	Beta^a^		–0.21	–0.16	0.31	0.11	–0.17	–0.28	–1.75	–0.52	0.60	0.71	1.37
	Normal	Cln{0°, 270°}	4.14	3.52	–1.90	–1.85	–	–	–	–	–30.10	38.76	12.30
	Beta		–0.08	0.11	0.53	0.02	0.82	0.49	–6.33	0.14	–3.62	15.15	–2.62
SD	Normal	BVN	1.84	2.68	1.59	1.74	24.50	14.06	28.18	17.58	24.52	27.58	25.93
	Beta		1.95	2.53	1.71	1.67	2.97	2.28	8.29	4.35	10.26	14.27	17.16
	Normal	Frank	1.89	2.74	1.65	1.81	24.70	14.04	28.44	17.80	24.90	28.57	26.45
	Beta		1.84	2.37	1.61	1.58	3.00	2.22	8.53	4.34	8.02	14.71	17.31
	Normal	Cln{180°, 90°}	1.88	2.67	1.62	1.73	23.97	13.53	27.78	17.86	23.67	25.46	21.90
	Beta^a^		1.98	2.52	1.68	1.67	2.85	2.15	8.89	4.28	9.18	14.65	15.85
	Normal	Cln{0°, 270°}	1.88	2.76	1.62	1.79	26.28	15.89	30.75	18.60	33.78	28.34	30.21
	Beta		1.98	2.63	1.74	1.71	3.59	2.83	9.05	4.54	16.13	16.23	19.53
$\sqrt{\bar{V}}$	Normal	BVN	1.38	2.39	1.17	1.66	16.86	10.85	25.40	14.73	15.55	15.62	15.66
	Beta		1.34	1.99	1.21	1.46	1.97	1.82	7.92	4.06	9.04	13.14	14.88
	Normal	Frank	1.31	2.28	1.12	1.62	16.21	10.76	24.94	14.66	13.13	13.75	14.50
	Beta		1.18	1.85	1.10	1.36	1.84	1.94	8.25	4.05	7.84	13.07	15.20
	Normal	Cln{180°, 90°}	1.36	2.34	1.15	1.63	16.49	10.37	24.44	14.14	13.51	15.53	13.77
	Beta^a^		1.33	1.99	1.21	1.44	1.92	1.83	8.00	3.97	8.08	13.45	14.33
	Normal	Cln{0°, 270°}	1.38	2.40	1.18	1.66	16.04	10.92	27.34	14.84	13.47	12.44	16.15
	Beta		1.22	1.85	1.10	1.36	2.10	1.94	7.84	4.14	10.83	12.75	16.73
RMSE	Normal	BVN	4.59	4.40	2.53	2.58	–	–	–	–	33.19	45.56	30.51
	Beta		1.95	2.53	1.75	1.67	2.97	2.28	9.58	4.35	11.42	15.56	17.28
	Normal	Frank	4.64	4.59	2.57	2.59	–	–	–	–	32.75	45.05	30.78
	Beta		1.85	2.41	1.61	1.59	3.00	2.23	9.53	4.35	8.43	15.58	17.42
	Normal	Cln{180°, 90°}	4.60	4.30	2.58	2.53	–	–	–	–	28.52	39.52	26.87
	Beta^a^		1.99	2.52	1.70	1.67	2.85	2.17	9.06	4.31	9.20	14.67	15.91
	Normal	Cln{0°, 270°}	4.55	4.47	2.50	2.58	–	–	–	–	45.24	48.01	32.62
	Beta		1.98	2.63	1.81	1.71	3.69	2.87	11.04	4.54	16.54	22.20	19.70

Note: Cln{$\omega_{1}^{{}^{\circ}},\omega_{2}^{{}^{\circ}}$}: The $C_{12}(\cdot ;\tau_{12}),C_{34}(\cdot ;\tau_{34})$ and $C_{23}(\cdot ;\tau_{23})$ pair-copulas are Clayton rotated by *ω*_1_ and *ω*_2_ degrees, respectively.

BVN: bivariate normal

a True model.

**Table 3.** Small sample of sizes *N *=* *30 simulations (10^3^ replications; *n_q_* = 15) from the multinomial quadrivariate D-vine copula mixed model with normal margins and resultant biases, root mean square errors (RMSE) and standard deviations (SD), along with the square root of the average theoretical variances ($\sqrt{\bar{V}}$), scaled by 100, for the ML estimates under different pair-copula choices and margins.

	Margin	Copula	$\pi_{1}$ = 0.94	$\pi_{2}$ = 0.79	$\pi_{3}$ = 0.03	$\pi_{4}$ = 0.09	$\sigma_{1}$ = 0.75	$\sigma_{2}$ = 0.65	$\sigma_{3}$ = 1.20	$\sigma_{4}$ = 0.69	$\tau_{12}$ = 0.82	$\tau_{23}$ = –0.38	$\tau_{34}$ = 0.29
Bias	Normal	BVN	–0.64	–0.33	0.61	0.25	0.99	–1.22	–5.03	–0.88	–6.98	4.30	5.50
	Beta		–6.16	–4.21	4.08	2.29	–	–	–	–	–15.26	–13.26	12.79
	Normal	Frank	–0.63	–0.17	0.61	0.22	0.82	–1.05	–5.73	–0.86	–6.67	2.53	5.45
	Beta		–5.97	–3.96	3.96	2.25	–	–	–	–	–12.55	–14.92	12.18
	Normal^a^	Cln{180°, 90°}	–0.63	–0.44	0.57	0.33	–1.13	–1.96	–2.71	–0.97	–1.54	–2.42	2.31
	Beta		–6.37	–4.42	4.10	2.50	–	–	–	–	–10.10	–19.62	9.71
	Normal	Cln{0°, 270°}	–0.72	–0.24	0.71	0.24	3.57	1.36	–3.63	–0.46	–4.08	11.78	4.52
	Beta		–6.20	–4.25	4.23	2.37	–	–	–	–	–21.47	–6.61	10.91
SD	Normal	BVN	2.12	2.75	1.83	1.84	18.29	11.62	23.06	14.40	17.54	17.42	19.35
	Beta		2.99	2.94	2.31	1.94	5.26	3.00	6.51	3.42	10.98	18.63	22.53
	Normal	Frank	2.20	2.80	1.91	1.88	17.92	11.60	23.42	14.50	14.46	18.54	20.16
	Beta		2.97	3.00	2.35	2.00	5.23	3.17	6.71	3.43	10.68	19.30	22.22
	Normal^a^	Cln{180°, 90°}	2.14	2.77	1.84	1.86	17.74	11.44	22.79	14.36	15.47	19.08	16.82
	Beta		3.06	3.03	2.34	2.01	5.16	3.25	7.07	3.50	11.46	20.35	21.01
	Normal	Cln{0°, 270°}	2.15	2.81	1.85	1.86	19.92	13.08	24.72	15.13	22.16	19.47	24.34
	Beta		2.99	3.02	2.33	1.98	5.73	3.39	6.60	3.46	16.13	21.83	30.25
$\sqrt{\bar{V}}$	Normal	BVN	1.43	2.45	1.19	1.62	15.81	10.23	22.66	12.43	18.18	15.88	15.91
	Beta		1.35	2.10	1.17	1.45	2.04	2.11	6.09	3.10	8.09	14.89	17.57
	Normal	Frank	1.33	2.30	1.11	1.55	15.53	10.13	22.28	12.37	11.75	15.14	16.00
	Beta		1.28	2.07	1.13	1.43	2.01	2.29	6.35	3.10	7.70	15.89	17.29
	Normal^a^	Cln{180°, 90°}	1.41	2.38	1.18	1.59	14.92	9.88	21.71	12.04	14.06	16.53	14.14
	Beta		1.31	2.14	1.17	1.45	1.97	2.29	6.58	3.13	7.93	14.92	16.86
	Normal	Cln{0°, 270°}	1.39	2.41	1.17	1.60	16.20	10.56	23.22	12.61	18.50	15.09	18.85
	Beta		1.26	1.95	1.08	1.34	2.20	2.09	5.72	3.15	8.89	18.63	20.45
RMSE	Normal	BVN	2.22	2.77	1.93	1.86	18.32	11.68	23.60	14.42	18.88	17.94	20.12
	Beta		6.85	5.13	4.69	3.00	–	–	–	–	18.80	22.87	25.91
	Normal	Frank	2.29	2.81	2.00	1.89	17.93	11.65	24.11	14.53	15.93	18.71	20.88
	Beta		6.67	4.96	4.61	3.01	–	–	–	–	16.48	24.39	25.34
	Normal^a^	Cln{180°, 90°}	2.23	2.80	1.93	1.89	17.77	11.61	22.95	14.39	15.55	19.24	16.98
	Beta		7.07	5.36	4.72	3.21	–	–	–	–	15.28	28.27	23.14
	Normal	Cln{0°, 270°}	2.27	2.82	1.98	1.88	20.24	13.15	24.98	15.13	22.53	22.75	24.76
	Beta		6.88	5.22	4.83	3.09	–	–	–	–	26.85	22.81	32.15

Note: Cln{$\omega_{1}^{{}^{\circ}},\omega_{2}^{{}^{\circ}}$}: The $C_{12}(\cdot ;\tau_{12}),C_{34}(\cdot ;\tau_{34})$ and $C_{23}(\cdot ;\tau_{23})$ pair-copulas are Clayton rotated by *ω*_1_ and *ω*_2_ degrees, respectively.

BVN: bivariate normal.

a True model.

Conclusions from the values in the tables are the following:

- ML with the true multinomial D-vine copula mixed model is highly efficient according to the simulated biases, SDs and RMSEs.
- The ML estimates of the univariate meta-analytic parameters and their SDs are robust under copula misspecification, but are not robust to margin misspecification.
- The ML estimates of *τ*’s and their SDs are robust to copula misspecification, but they are not robust to margin misspecification.

### 3.2 Misspecification of the copula mixed model that accounts for non-evaluable outcomes

We randomly generate samples of size *N *=* *30 from the multinomial quadrivariate D-vine copula mixed model and the extended trivariate vine copula mixed model with both normal and beta margins using the algorithm in Section 3.1 and in Nikoloulopoulos,^6^ respectively. We compare the ML estimates of common parameters for both approaches under misspecification and also include in the comparison the bivariate copula mixed model estimates where the non-evaluable positives and negatives are either excluded or included as false negatives and false positives (intention to diagnose approach), respectively.

In Section 3.1 and in Nikoloulopoulos,^6^ it has been revealed that (a) the estimation of the univariate meta-analytic parameters is a univariate inference, and hence it is the univariate marginal distribution that matters and not the type of the copula, and (b) estimated Kendall’s *τ* is similar among different families of copulas. Hence, as the ML estimates are nearly not affected by the type of the pair-copula, we provide here the results when all the bivariate copulas are BVN.

Tables 4 and 5 contain the resultant biases, RMSEs, and SDs, along with the square root of the average theoretical variances ($\sqrt{\bar{V}}$), scaled by 100, for the ML estimates under different copula mixed models. The true quadrivariate multinomial vine copula mixed model is composed by the Clayton copulas rotated by $180^{{}^{\circ}}$ for both the $C_{12}(;\tau_{12})$ and $C_{34}(;\tau_{34})$ pair-copulas and the Clayton copula rotated by $90^{{}^{\circ}}$ for the $C_{23}(;\tau_{23})$ pair-copula. The true trivariate vine copula mixed model is composed by the Clayton copula for $C_{12}(;\tau_{12})$ and the Clayton rotated by $90^{{}^{\circ}}$ for both the $C_{13}(;\tau_{13})$ and $C_{23|1}(;\tau_{23|1})$ pair-copulas.

**Table 4.** Small sample of sizes *N *=* *30 simulations (10^3^ replications; *n_q_* = 15) from the multinomial quadrivariate D-vine and trivariate vine copula mixed models with beta margins and resultant biases, root mean square errors (RMSE) and standard deviations (SD), along with the square root of the average theoretical variances ($\sqrt{\bar{V}}$), scaled by 100, for the ML estimates under different copula mixed models.

			True vine copula mixed model									
			Trivariate					Quadrivariate				
	Fitted copulamixed model	Margin	$\pi_{1}$ = 0.97	$\pi_{2}$ = 0.85	$\gamma_{1}$ = 0.03	$\gamma_{2}$ = 0.06	τ = 0.39	$\pi_{1}$ = 0.90	$\pi_{2}$ = 0.77	$\gamma_{1}$ = 0.09	$\gamma_{2}$ = 0.08	τ = 0.82
Bias	Bivariate^a^	Beta	0.04	0.22	–0.11	–0.15	11.37	7.10	9.63	–5.84	–2.25	–42.16
		Normal^b^	0.91	2.38	–	–	14.24	8.26	11.78	–	–	–40.39
	Bivariate^c^	Beta	–3.18	–5.46	1.70	0.14	–2.54	–0.08	–0.03	–0.08	–0.22	–4.75
		Normal^b^	–1.59	–3.50	–	–	–1.26	2.79	1.47	–	–	–2.35
	Trivariate	Beta	–0.03	–0.09	–0.10	–0.06	8.97	7.10	9.60	–5.81	–2.24	–42.47
		Normal^d^	0.86	2.13	–	–	10.89	8.25	11.76	–	–	–40.61
	Quadrivariate	Beta	–3.18	–5.46	1.71	0.13	–1.63	–0.08	–0.03	–0.10	–0.21	–5.01
		Normal	–0.46	–1.10	–	–	21.73	4.20	3.49	–	–	–22.37
SD	Bivariate^a^	Beta	0.64	1.91	1.29	2.00	24.08	0.81	1.83	1.91	1.99	17.07
		Normal^b^	0.57	1.90	23.49	14.40	25.41	0.49	1.72	23.15	13.93	17.40
	Bivariate^c^	beta	0.77	1.85	1.58	1.92	17.45	1.94	2.54	2.94	2.22	10.03
		Normal^b^	0.80	1.94	19.38	12.22	18.29	1.87	2.86	25.11	14.07	9.06
	Trivariate	Beta	0.66	1.89	1.27	2.02	22.71	0.81	1.83	1.93	1.99	16.92
		Normal^d^	0.58	1.88	23.45	14.46	23.79	0.48	1.72	23.51	14.10	17.35
	Quadrivariate	Beta	0.77	1.87	1.57	1.93	18.20	1.95	2.53	2.97	2.28	10.25
		Normal	0.68	1.91	23.35	13.83	23.92	1.84	2.68	24.50	14.06	24.90
$\sqrt{\bar{V}}$	Bivariate^a^	Beta	0.63	1.80	1.34	2.02	27.90	0.60	1.61	1.21	1.69	15.20
		Normal^b^	0.53	1.73	24.78	14.05	26.16	0.45	1.52	19.40	11.91	15.35
	Bivariate^c^	beta	1.08	2.09	1.78	2.00	16.39	1.31	1.97	1.93	1.79	8.79
		Normal^b^	0.93	2.10	19.55	11.99	17.09	1.27	2.04	16.09	10.47	8.03
	Trivariate	Beta	0.66	1.86	1.37	2.06	25.75	0.60	1.60	1.20	1.68	15.06
		Normal^d^	0.54	1.77	24.03	14.24	22.36	0.45	1.52	19.13	11.82	15.12
	Quadrivariate	Beta	1.09	2.09	1.78	2.00	17.08	1.34	1.99	1.97	1.82	9.04
		Normal	0.81	2.01	24.81	13.61	22.07	1.38	2.39	16.86	10.85	15.55
RMSE	Bivariate^a^	Beta	0.64	1.92	1.29	2.00	26.63	7.15	9.80	6.14	3.00	45.48
		Normal^b^	1.08	3.04	–	–	29.13	8.28	11.91	–	–	43.98
	Bivariate^c^	Beta	3.27	5.77	2.32	1.92	17.64	1.94	2.54	2.94	2.23	11.10
		Normal^b^	1.78	4.01	–	–	18.34	3.36	3.21	–	–	9.36
	Trivariate	Beta	0.67	1.89	1.28	2.02	24.42	7.15	9.77	6.12	3.00	45.72
		Normal^d^	1.04	2.84	–	–	26.16	8.27	11.88	–	–	44.16
	Quadrivariate	Beta	3.27	5.77	2.32	1.94	18.27	1.95	2.53	2.97	2.28	11.42
		Normal	0.82	2.21	–	–	32.32	4.59	4.40	–	–	33.19

a The non-evaluable outcomes are excluded

b The resulting model is the same as the BGLMM.

c The non-evaluable positives and negatives are included as false negatives and positives, respectively.

d The resulting model is the same as the extended TGLMM.

**Table 5.** Small sample of sizes *N *=* *30 simulations (10^3^ replications; *n_q_* = 15) from the multinomial quadrivariate D-vine and trivariate vine copula mixed models with normal margins and resultant biases, root mean square errors (RMSE) and standard deviations (SD), along with the square root of the average theoretical variances ($\sqrt{\bar{V}}$), scaled by 100, for the ML estimates under different copula mixed models.

			True vine copula mixed model									
			Trivariate					Quadrivariate				
	Fitted copulamixed model	Margin	$\pi_{1}$ = 0.98	$\pi_{2}$ = 0.88	$\sigma_{1}$ = 0.90	$\sigma_{2}$ = 0.73	τ = 0.39	$\pi_{1}$ = 0.94	$\pi_{2}$ = 0.79	$\sigma_{1}$ = 0.75	$\sigma_{2}$ = 0.65	τ = 0.82
Bias	Bivariate^a^	Beta	–0.85	–1.91	–	–	13.57	2.69	6.59	–	–	–4.63
		Normal^b^	–0.03	0.17	–6.52	–1.39	15.81	3.35	8.18	–1.80	–1.96	–4.69
	Bivariate^c^	Beta	–4.04	–7.58	–	–	–1.43	–5.97	–3.98	–	–	–17.33
		Normal^b^	–2.46	–5.61	–0.79	–6.12	–0.35	–1.75	–1.93	63.85	17.11	–14.39
	Trivariate	Beta	–0.92	–2.19	–	–	10.97	2.63	6.45	–	–	–7.20
		Normal^d^	–0.08	–0.07	–6.28	–1.15	12.26	3.31	8.09	–1.64	–2.09	–7.31
	Quadrivariate	Beta	–4.04	–7.57	–	–	–0.13	–6.16	–4.21	–	–	–15.26
		Normal	–1.39	–3.28	–8.23	–3.69	23.31	–0.64	–0.33	0.99	–1.22	–6.98
SD	Bivariate^a^	Beta	0.69	1.94	1.43	2.13	25.07	0.54	1.61	0.80	1.56	17.37
		Normal^b^	0.52	1.75	24.43	13.65	26.48	0.44	1.48	17.34	11.50	16.85
	Bivariate^c^	Beta	0.78	1.83	1.54	1.94	17.78	2.95	2.86	5.50	3.08	10.51
		Normal^b^	0.76	1.84	18.23	11.72	18.50	2.11	2.96	25.41	13.97	9.93
	Trivariate	Beta	0.72	1.92	1.43	2.15	23.29	0.60	1.69	0.86	1.55	17.14
		Normal^d^	0.54	1.73	24.00	13.63	24.34	0.47	1.55	17.87	11.40	16.62
	Quadrivariate	Beta	0.79	1.84	1.53	1.96	18.77	2.99	2.94	5.26	3.00	10.98
		Normal	0.66	1.78	24.11	13.34	25.91	2.12	2.75	18.29	11.62	17.54
$\sqrt{\bar{V}}$	Bivariate^a^	Beta	0.59	1.75	1.18	1.93	29.79	0.50	1.48	0.71	1.38	19.52
		Normal^b^	0.50	1.67	23.60	13.40	30.27	0.45	1.45	16.23	10.60	23.19
	Bivariate^c^	Beta	1.06	2.06	1.71	1.97	16.68	1.33	2.10	2.01	2.13	8.30
		Normal^b^	0.91	2.07	18.92	11.82	16.94	1.34	2.22	15.15	11.02	8.07
	Trivariate	Beta	0.61	1.78	1.20	1.94	25.49	0.51	1.48	0.72	1.37	18.94
		Normal^d^	0.51	1.70	22.86	13.25	22.97	0.45	1.45	15.63	10.37	19.41
	Quadrivariate	Beta	1.06	2.07	1.70	1.98	16.83	1.35	2.10	2.04	2.11	8.09
		Normal	0.79	1.96	22.00	12.84	24.51	1.43	2.45	15.81	10.23	18.18
RMSE	Bivariate^a^	Beta	1.10	2.72	–	–	28.51	2.74	6.79	–	–	17.98
		Normal^b^	0.52	1.75	25.28	13.72	30.84	3.38	8.31	17.43	11.66	17.49
	Bivariate^c^	Beta	4.11	7.80	–	–	17.83	6.66	4.90	–	–	20.27
		Normal^b^	2.58	5.91	18.25	13.22	18.50	2.74	3.53	68.73	22.09	17.49
	Trivariate	Beta	1.17	2.91	–	–	25.74	2.70	6.67	–	–	18.59
		Normal^d^	0.55	1.73	24.81	13.67	27.25	3.35	8.23	17.94	11.59	18.16
	Quadrivariate	Beta	4.12	7.79	–	–	18.77	6.85	5.13	–	–	18.80
		Normal	1.54	3.73	25.47	13.84	34.85	2.22	2.77	18.32	11.68	18.88

a The non-evaluable outcomes are excluded.

b The resulting model is the same as the BGLMM.

c The non-evaluable positives and negatives are included as false negatives and positives, respectively.

d The resulting model is the same as the extended TGLMM.

Conclusions from the values in the tables are the following:

- The bivariate copula mixed model where the non-evaluable outcomes are disregarded and the extended trivariate vine copula mixed model showed similar performance. Both led to unbiased (biased) and efficient (inefficient) estimates when the true model is the trivariate (quadrivariate multinomial) vine copula mixed model.
- The bivariate copula mixed model where the non-evaluable positives and negatives included as false negatives and false positives, respectively, and the multinomial D-vine copula mixed model with beta margins showed similar performance. Both led to unbiased (biased) and efficient (inefficient) estimates when the true model is the quadrivariate multinomial vine copula mixed model with beta margins (trivariate vine copula mixed model or quadrivariate multinomial vine copula mixed model with normal margins).

## 4 Meta-analysis of coronary computed tomography angiography studies

We apply the multinomial quadrivariate D-vine copula mixed model for the meta-analysis of diagnostic accuracy studies accounting for non-evaluable subjects to data on 30 studies from a systematic review for diagnostic accuracy studies of coronary computed tomography angiography for the detection of coronary artery disease.^27^

We fit the multinomial quadrivariate D-vine copula mixed model for all different decompositions of the D-vine copula density, for both beta and normal margins and different pair-copulas at the level 1; for levels 2 and 3, we use BVN copulas. In cases when fitting the multinomial quadrivariate D-vine copula mixed model, the resultant estimate of one of the conditional dependence parameters was close to the right boundary of its parameter space (that is clear indication that the model with a full structure provides more dependence structure than it is actually required^8^), we used a truncated model, i.e. we captured the strongest dependence in the first tree and then just used the independence copulas in lower order trees, i.e. conditional independence. Joe et al.^28^ showed that in order for a vine copula to have (tail) dependence for all bivariate margins, it is only necessary for the bivariate copulas in level 1 to have (tail) dependence and it is not necessary for the conditional bivariate copulas in levels 2 and 3, to have tail dependence. Hence, one can either use BVN or independence copulas at levels 2 and 3 without sacrificing the tail dependence of the vine copula distribution.

In Table 6, we present the results from the decomposition of the vine copula density in equation (4), as a different decompositions led to similar results due to the small sample size. This is consistent with our previous studies on vine copula mixed models.^6,8^ Since the number of parameters is not the same between the models, we use the Akaike information criterion (AIC), that is −2× log-likelihood +2× (#model parameters) as a rough diagnostic measure for goodness of fit between the models. The AICs showed that a (truncated) multinomial quadrivariate D-vine copula mixed model with Clayton copulas rotated by 180° for both the $C_{12}(;\tau_{12})$ and $C_{34}(;\tau_{34})$ pair-copulas and the Clayton copula rotated by 90° for the $C_{23}(;\tau_{23})$ pair-copula and beta margins (Table 6) provides the best fit.

**Table 6.** AICs, ML estimates, and standard errors (SE) of the multinomial quadrivariate D-vine copula mixed models for diagnostic accuracy studies of coronary computed tomography angiography.

	BVN		Frank		Cln{180°, 90°}^a^		Cln{180°, 270°}	
	Est.	SE	Est.	SE	Est.	SE	Est.	SE
Normal margins								
*π*_1_	0.94	0.01	0.95	0.01	0.94	0.02	0.94	0.02
*π*_2_	0.80	0.03	0.80	0.03	0.79	0.03	0.79	0.03
*π*_3_	0.04	0.01	0.03	0.01	0.03	0.01	0.04	0.01
*π*_4_	0.09	0.02	0.09	0.02	0.09	0.02	0.09	0.02
*σ*_1_	0.89	0.20	0.91	0.19	0.75	0.17	0.83	0.17
*σ*_2_	0.72	0.15	0.65	0.13	0.65	0.12	0.67	0.13
*σ*_3_	1.32	0.36	1.37	0.36	1.20	0.31	1.19	0.33
*σ*_4_	0.80	0.23	0.70	0.21	0.69	0.19	0.73	0.19
*τ*_12_	0.54	0.22	0.49	0.20	0.82	0.19	0.82	0.18
*τ*_23_	–0.16	0.20	–0.31	0.17	–0.38	0.24	–0.04	0.15
*τ*_34_	0.22	0.23	0.11	0.24	0.29	0.17	0.37	0.17
$\tau_{13|2}$	0.43	0.34	0.67	0.23	–	–	–	–
$\tau_{24|3}$	0.11	0.22	–0.03	0.24	–	–	–	–
$\tau_{14|23}$	–0.39	0.32	–0.36	0.49	–	–	–	–
AIC	4013.22		4010.80		4007.72		4009.36	
Beta margins								
*π*_1_	0.90	0.02	0.90	0.02	0.90	0.01	0.89	0.01
*π*_2_	0.76	0.03	0.77	0.02	0.77	0.02	0.76	0.02
*π*_3_	0.06	0.01	0.06	0.01	0.06	0.01	0.07	0.01
*π*_4_	0.11	0.02	0.11	0.02	0.11	0.02	0.11	0.02
*γ*_1_	0.08	0.03	0.09	0.03	0.09	0.03	0.10	0.03
*γ*_2_	0.09	0.03	0.09	0.02	0.08	0.02	0.09	0.02
*γ*_3_	0.32	0.12	0.32	0.13	0.37	0.12	0.28	0.12
*γ*_4_	0.15	0.07	0.16	0.07	0.15	0.07	0.15	0.06
*τ*_12_	0.71	0.11	0.74	0.08	0.82	0.08	0.79	0.07
*τ*_23_	–0.35	0.17	–0.34	0.12	–0.52	0.14	–0.23	0.10
*τ*_34_	0.23	0.22	0.20	0.21	0.26	0.18	0.21	0.17
$\tau_{13|2}$	–0.66	0.38	–	–	–	–	–	–
$\tau_{24|3}$	–0.10	0.20	–	–	–	–	–	–
$\tau_{14|23}$	–0.02	0.57	–	–	–	–	–	–
AIC	4009.42		4005.93		4002.17		4004.92	

Note: Cln{$\omega_{1}^{{}^{\circ}},\omega_{2}^{{}^{\circ}}$}: The $C_{12}(\cdot ;\tau_{12}),C_{34}(\cdot ;\tau_{34})$ and $C_{23}(\cdot ;\tau_{23})$ pair-copulas are Clayton rotated by *ω*_1_ and *ω*_2_ degrees, respectively.

AIC: akaike information criterion; BVN: bivariate normal.

a Best fit.

In real data (in contrast with the simulated data in Section 3), the truth is unknown, so it is important to compare between the proposed and other existing approaches in terms of point estimation and variance. First, in order to reveal if the use of the proposed model is worthy, when a standard bivariate analysis (either ignoring the non-evaluable outcomes or including the non-evaluable positives and negatives as false negatives and positives, respectively) is easy, we also fit the bivariate copula mixed model^10^ with both beta and normal margins and different bivariate copulas. According to the likelihood principle, a bivariate copula mixed model with a Clayton and Clayton copula rotated by 180° (to model the association between the latent sensitivity and specificity) and beta margins provides the best fit for both different ad-hoc approaches to handle the non-evaluable outcomes (Table 7). It is revealed that a bivariate copula mixed model with the sensitivity and specificity on the original scale provides better fit than the BGLMM,^7^ which models the sensitivity and specificity on a transformed scale.

**Table 7.** AICs, ML estimates, and standard errors (SE) of the best fitted bivariate copula and extended trivariate vine copula mixed models with beta margins for diagnostic accuracy studies of coronary computed tomography angiography.

	Bivariate				Trivariate	
	Clayton^a^		Clayton 180°^b^		Clayton {0°,90°}	
	Est.	SE	Est.	SE	Est.	SE
*π*_1_	0.97	0.01	0.90	0.01	0.97	0.01
*π*_2_	0.85	0.02	0.77	0.02	0.85	0.02
*π*_3_	–	–	–	–	0.49	0.03
*γ*_1_	0.03	0.01	0.09	0.03	0.03	0.01
*γ*_2_	0.06	0.02	0.08	0.02	0.06	0.02
*γ*_3_	–	–	–	–	0.11	0.02
*τ*_12_	0.42	0.19	0.82	0.08	0.39	0.20
*τ*_13_	–	–	–	–	0.02	0.23
$\tau_{23|1}$	–	–	–	–	–0.28	0.17
AIC	244.82		321.91		492.26	

AIC: akaike information criterion.

a The non-evaluable outcomes are excluded.

b The non-evaluable positives and negatives are included as false negatives and positives, respectively.

Then, in order to compare the proposed approach with the ones that use the MAR assumption, we fit the extended trivariate vine copula mixed model^6^ with both beta and normal margins and different pair-copulas. According to the likelihood principle, a vine copula mixed model composed of a Clayton copula to model the association between the sensitivity and specificity, a Clayton copula rotated by 90° to model both the associations between the specificity and prevalence and between the sensitivity and prevalence given the specificity, and beta margins provides the best fit (Table 7). It is revealed that an extended trivariate vine copula mixed model with the sensitivity, specificity, and disease prevalence on the original scale provides better fit than the extended TGLMM,^5^ which models the sensitivity, specificity, and disease prevalence on a transformed scale.

It has been shown that the trivariate analysis does not change the conclusions from the bivariate analysis excluding the non-evaluable outcomes. It is also apparent that the results from the quadrivariate analysis differentiate from the ones from bivariate (excluding the non-evaluable outcomes) and trivariate analyses which are fairly similar. The meta-analytic estimates of sensitivity and specificity from the latter approaches are blown, because in both approaches it is assumed that

$$Y_{i11}|X_{1}=x_{1}∼\text{Binomial}\left(y_{i01}+y_{i11},\;x_{1}\right)\;\text{ and }\;Y_{i00}|X_{2}=x_{2}∼\text{Binomial}\left(y_{i00}+y_{i10},\;x_{2}\right)$$

i.e. their support ignores the number of non-evaluable positives $y_{i21}$ and the number of non-evaluable negatives $y_{i20}$. The conclusions from the quadrivariate analysis with the latent proportions on the original scale are quite similar with the ones from the bivariate analysis where the non-evaluable positives and negatives are included as false negatives and positives, respectively. These results are consistent with the findings in the simulations in Section 3.2. Note in passing that comparing the AIC values among the quadrivariate, trivariate and bivariate copula mixed models is inconclusive as they use a different number of responses.

Although typically the focus of meta-analysis has been to derive the summary-effect estimates, there is increasing interest in drawing predictive inference. Summary receiver operating characteristic curves (SROC) can be deduced from the D-vine copula mixed model with the sensitivity and specificity on the original scale through the quantile regression techniques developed for the bivariate copula mixed model.^10^ SROC essentially shows the effect of different model (random effect distribution) assumptions, since it is an inference that depends on the joint distribution. An SROC curve has been deduced for the bivariate copula mixed model^10^ through a median regression curve of *X*_1_ on *X*_2_. For the copula mixed model, the model parameters (including dependence parameters), the choice of the copula, and the choice of the margin affect the shape of the SROC curve.^10^ However, there is no priori reason to regress *X*_1_ on *X*_2_ instead of the other way around, so a median regression curve of *X*_2_ on *X*_1_ has also been provided. Rucker and Schumacher^29^ stated that instead of summarizing data using an SROC, it might be preferable to give confidence regions. Hence, in addition to using just median regression curves, quantile regression curves with a focus on high (*q *=* *0.99) and low quantiles (*q *=* *0.01), which are strongly associated with the upper and lower tail dependence imposed from each parametric family of copulas, have been proposed.^10^ These can been seen as confidence regions of the median regression SROC curve.

**Figure 1.**
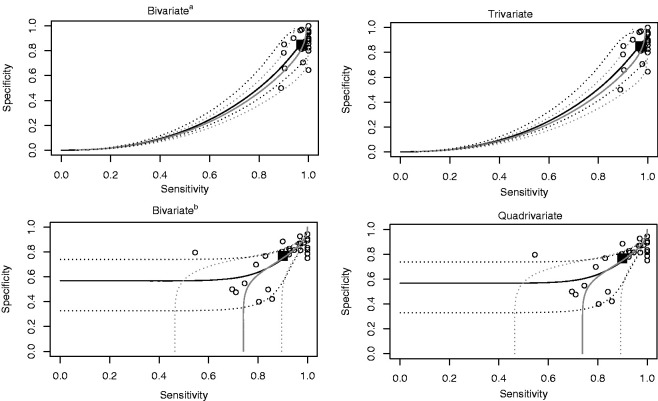
SROC curves with a confidence region and summary operating points (a pair of the model-based sensitivity and specificity) from the best fitted multinomial quadrivariate D-vine, extended trivariate vine and bivariate copula mixed models along with the study estimates. ■: summary point; °: study estimate; black and grey lines represent the quantile regression curves $x_{1}:={\tilde{x}}_{1}(x_{2},q)$ and $x_{2}:={\tilde{x}}_{2}(x_{1},q)$, respectively; for *q* = 0.5 solid lines and for q∈{0.01,0.99} dotted lines (confidence region).^a^The non-evaluable outcomes are excluded.^b^The non-evaluable positives and negatives are included as false negatives and positives, respectively.

Figure 1 demonstrates the SROC curves with a confidence region and summary operating points (a pair of the model-based sensitivity and specificity; shown by the black square) from the best fitted multinomial quadrivariate D-vine copula mixed model, the best fitted trivariate vine copula mixed model, and the best fitted bivariate copula mixed models along with the study estimates (shown by the circles in Figure 1). For the upper panel graphs, the sensitivity and specificity at study *i* (point estimates) have been calculated with the typical definitions of sensitivity and specificity, viz.

$$\frac{y_{i11}}{y_{i01}+y_{i11}}\;\text{ and }\;\frac{y_{i00}}{y_{i00}+y_{i10}}$$

respectively, as only patients with positive or negative results are considered, while for the lower panel graphs, the sensitivity and specificity at study *i* have been calculated with the definitions of sensitivity and specificity in Simel et al.,^30^ viz.

$$\frac{y_{i11}}{y_{i+1}}\;\text{ and }\;\frac{y_{i00}}{y_{i+0}}$$

respectively, since the number of non-evaluable positives $y_{i21}$ contributes to the diseased population and the number of non-evaluable negatives $y_{i20}$ contributes to the non-diseased population.

## 5 Discussion

Motivated by the existence of non-evaluable results in diagnostic test accuracy studies, this paper proposed a multinomial quadrivariate D-vine copula mixed model for meta-analysis of diagnostic test accuracy studies accounting for non-evaluable subjects. Our general statistical model allows for selection of pair-copulas independently among a variety of parametric copula families, i.e. there are no constraints in the choices of bivariate parametric families of copulas and can also operate on the original scale of sensitivity and specificity.

For the random effects, we have used a quadrivariate D-vine copula distribution or a truncated at level 1 quadrivariate D-vine copula (conditional independence), which allows flexible (tail) dependence.^28^ We have proposed a numerically stable ML estimation technique based on Gauss-Legendre quadrature; the crucial step is to convert from independent to dependent quadrature points that follow a quadrivariate D-vine distribution.

In an era of evidence-based medicine, decision makers need high-quality procedures such as the one developed in this article to support decisions about whether or not to use a diagnostic test in a specific clinical situation. The multinomial quadrivariate vine-copula mixed model is not an ad-hoc^2^ but rather a sophisticated approach that utilizes all the available data in decision making and can satisfy the intention-to-diagnose principle. Using an intention to diagnose principle, i.e. a conservative approach, ensures that both the sensitivity and specificity are not overestimated. Hence, it formally enables decision makers to be more cautious in solely relying to the overly optimistic meta-analytic estimates of sensitivity and specificity derived from the extended trivariate vine copula mixed model that indirectly accounts for the non-evaluable outcomes.

## Software

R functions to implement and simulate from the multinomial quadrivariate D-vine copula mixed model for meta-analysis of diagnostic tests with non-evaluable subjects are part of the R package CopulaREMADA.^31^ The data and code used in Section 4 are given as data and code examples in the package, respectively.
